# An Iterative, Mixed Usability Approach Applied to the Telekit System from the Danish TeleCare North Trial

**DOI:** 10.1155/2016/6351734

**Published:** 2016-11-16

**Authors:** Pernille Heyckendorff Lilholt, Clara Schaarup, Ole Kristian Hejlesen

**Affiliations:** Department of Health Science and Technology, Aalborg University, Aalborg, Denmark

## Abstract

*Objective*. The aim of the present study is to evaluate the usability of the telehealth system, coined Telekit, by using an iterative, mixed usability approach.* Materials and Methods*. Ten double experts participated in two heuristic evaluations (HE1, HE2), and 11 COPD patients attended two think-aloud tests. The double experts identified usability violations and classified them into Jakob Nielsen's heuristics. These violations were then translated into measurable values on a scale of 0 to 4 indicating degree of severity. In the think-aloud tests, COPD participants were invited to verbalise their thoughts.* Results*. The double experts identified 86 usability violations in HE1 and 101 usability violations in HE2. The majority of the violations were rated in the 0–2 range. The findings from the think-aloud tests resulted in 12 themes and associated examples regarding the usability of the Telekit system. The use of the iterative, mixed usability approach produced both quantitative and qualitative results.* Conclusion*. The iterative, mixed usability approach yields a strong result owing to the high number of problems identified in the tests because the double experts and the COPD participants focus on different aspects of Telekit's usability. This trial is registered with Clinicaltrials.gov, NCT01984840, November 14, 2013.

## 1. Introduction

Information technology has developed exponentially in recent years, influencing substantially the field of telehealth. Thus, the outcomes of trials of telehealth systems have demonstrated that telehealth can provide economic gains, superior continuity of care, and more self-sufficient patients [1–3]. The Danish cluster-randomised, controlled, large-scale trial, TeleCare North, is an example hereof. TeleCare North was implemented in 2014-2015, recruiting a total of 1,225 patients from the North Denmark Region. The purpose of this trial was to assess the effectiveness and cost-effectiveness of a telehealth system (named Telekit) designed for patients with chronic obstructive pulmonary disease (COPD) compared with usual practice. In Denmark,* usual practice* includes care, monitoring, and treatment of patients, and these tasks are performed by general practitioners and municipality healthcare workers such as community nurses. The purpose of the Telekit system is to allow COPD patients to gain more insight into their own disease and to support their skills and resources in relation to the management of their disease, thereby giving them greater control over their lives and hopefully enhancing their quality of life. The outcomes of the TeleCare North trial include changes in quality of life, mortality, physiological indicators, and the incremental cost-effectiveness ratio measured from baseline to follow-up at 12 months [4].

Self-management initiatives such as the TeleCare North trial are increasingly being integrated into healthcare as self-management initiatives are implemented to reduce admission rates, improve quality of life, and prevent worsening of the patient's condition. Despite the growing popularity of self-management initiatives, people encountering problems using systems commonly stop using the technologies or withdraw early from studies [5]. Identifying patient factors explaining this behaviour, extant literature suggests that many existing systems are directed towards clinical users and focus less on pertinent patient factors like technology needs, capabilities, and psychological and environmental barriers, and so forth. Even when patient factors are taken into account, they are often prioritized only when the user interface is prepared and less when the system is being conceived and designed [6].

Developing and implementing a system do not require attention only to the technical requirements. The processes also depend on user involvement and participation in the form of, for example, user satisfaction, usability, and a recognized need for the technology in daily life. The users' interaction with the telehealth technologies is therefore particularly important because their level of interaction shows whether they want to make use of the telehealth technologies or prefer opting out. Many patients using telehealth are elderly patients who suffer from chronic disease, have limited skills, and are socioeconomically disadvantaged. These limitations need to be considered throughout the design process of telehealth technologies [7, 8]. Patient safety is another important aspect because telehealth technologies involve transmitting information and communicating with patients. The healthcare professionals need to be sure that data are transmitted securely and that only relevant healthcare professionals have access to the transmitted data. To provide patient outcomes and clinical outcomes that are satisfactory, telehealth technologies need to show good usability [9].

Usability is defined as follows by the International Organisation for Standardisation (ISO 9241-11):

*The extent to which a product can be used by specified users to achieve specified goals with effectiveness, efficiency and satisfaction in a specified context of use [10]. *

Several different usability testing methods are available for collecting user perspectives with a view to improving the usability of telehealth technologies [11]. Usability testing measures the ease of use of a technology quantitatively or qualitatively. The literature on the topic indicates that several different methods should be used when testing usability as each method has strengths and limitations and provides different perspectives on usability [12–16]. The literature on this topic highlights the importance of using mixed usability methods iteratively. Iteration refers to the process by which development activities are repeated or looped during system development and how each loop is revised. Iteration, therefore, means going through multiple design versions of a system by conducting usability evaluations and revising the system based on the usability findings made [14, 15]. Usability expert Jakob Nielsen argues in favour of iterative usability testing; specifically, he recommends two iterations (one iteration is a redesign of two design versions) or more, because the first redesign will have many remaining problems [17].

Using an iterative, mixed usability approach, the purpose of this article was to evaluate the usability of the Telekit system from the Danish TeleCare North trial with a view to improving its quality and functionality. The usability methods were two heuristic evaluations (HE1, HE2) and two think-aloud tests (TA1, TA2). The usability evaluations were divided into a pretest and a posttest: (1) the pretest included usability evaluations (HE1, TA1) and was performed on two consecutive versions of the Telekit system in two previous studies [18, 19]. HE1 was performed early in Telekit's design process to assess potential usability problems that could complicate the implementation of the system. The problems found in HE1 have triggered several substantial changes and a number of updated versions of the Telekit system [17, 18]. After the heuristic evaluation (HE1), a think-aloud test (TA1) was performed to determine the users' experiences with the revised version of the Telekit system [20]. (2) The posttest included usability evaluations (HE2, TA2) performed after completion of the TeleCare North trial. Specifically, a second heuristic evaluation (HE2) and a second think-aloud test (TA2) were performed on the latest, updated version of the Telekit system.

Various evaluation methods and strategies have already been developed, and several studies have used different iterative usability testing methods in combination [20, 21]. The goal of this study was to evaluate the usability of the Telekit system through iterative, mixed usability testing. Quantitative results and qualitative findings were generated and these were compared in order to assess the usability of the Telekit system.

## 2. Materials and Methods

### 2.1. Study Population

The study population consisted of two different groups of participants. One group (*n* = 10) attended the heuristic evaluations (HE1, HE2) and another (*n* = 11) attended the think-aloud tests (TA1, TA2). All participants received verbal or written information about the study procedure and consented to participate in the study.

#### 2.1.1. Study Population—Heuristic Evaluations (HE1, HE2)

Five usability experts were invited to participate in each heuristic evaluation. Some experts (experts number (1) and number (3)) took part in both evaluations. All experts were biomedical engineers and were serving as Ph.D. fellows or associate/assistant professors with the Department of Health Science and Technology, Aalborg University, Denmark. They all had specific expertise in the health domain and in usability and the user interface being evaluated, which made it reasonable to denote them as double experts.

#### 2.1.2. Study Population—Think-Aloud Test (TA1)

Six participants (three men, three women) with COPD attended TA1 [20]. They were randomly selected among 17 pilot patients from the Danish TeleCare North trial [4]. Their average age was 69 years (min 65, max 73), and they represented different stages of COPD (one mild, one moderate, and four severe). The participants in the severe stage needed oxygen therapy. Their technology experiences varied; some were novices, some were knowledgeable, and some were daily users. The participants had 6 months of experience with the Telekit system before taking the TA1.

#### 2.1.3. Study Population—Think-Aloud Test (TA2)

Five participants (one man, four women) with COPD were recruited from the Danish TeleCare North trial [4]. We contacted the district nurses from the trial who then randomly selected potential users. Their average age was 65 years (min 50, max 72); and the participants had previously worked as childminder (*n* = 1), grocer (*n* = 2), housewife (*n* = 1), and a sandwich preparer (*n* = 1). Two participants had moderate COPD (lung capacity in the 50–80% range) and three had severe COPD (lung capacity 30–55%). Those in the severe stage of the disease were wheelchair users and required oxygen therapy.

Inclusion criteria were outlined before recruitment to the think-aloud test was initiated:

- Participants should have recently received the Telekit system (<3 months) because they had to maintain their curiosity and should not have developed habits in the use of the Telekit system.
- Participants should have received the first of two training sessions before initiating the think-aloud test. This was important because they needed basic knowledge about the use of the Telekit system; otherwise, they would not be able to use the system during the test.
- Participants should be fairly well functioning in order to be able to complete the think-aloud test. Thus, only patients who were able to express their experiences with the Telekit system could be included.

### 2.2. The Telekit System

The Telekit system was developed by Silverbullet A/S [22, 23] and was designed to support patients diagnosed with COPD, diabetes, and heart failure in the management of their disease, and also to reduce the healthcare costs associated with these diseases. Telekit operates on an open source platform, OpenTele, which is also used in two other Danish regions, besides the North Denmark Region. The OpenTele platform is a license-free platform that collects the patient's health data and health professionals' interaction with data and transports data to central databases [22].

**Figure 1:**
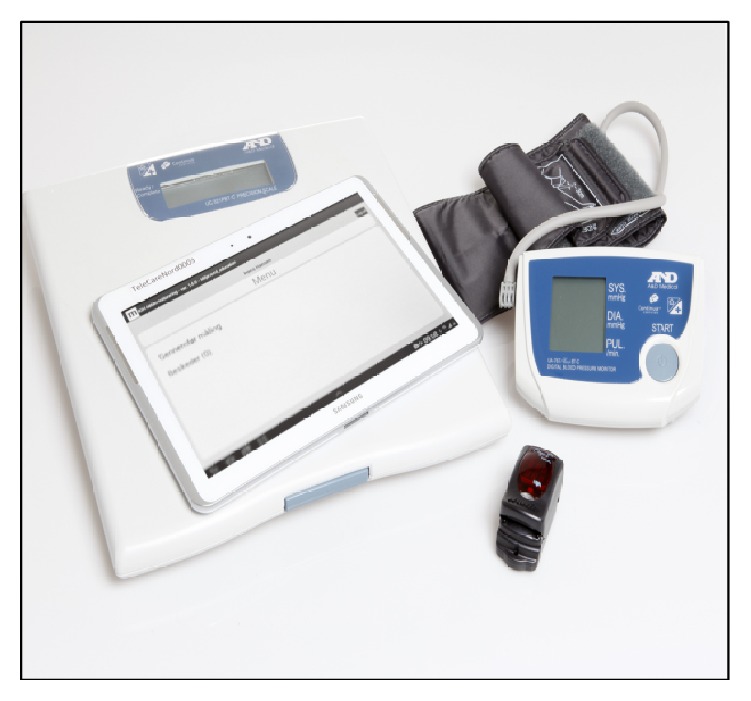
The Telekit system from the Danish TeleCare North trial. The COPD patients use Telekit to measure their vital signs.

The COPD patients receive instructions for the use of Telekit by community nurses either at home or at a healthcare center. Instructions are given as two training sessions. The Telekit system consists of a small portable carrying case containing a tablet (Samsung Galaxy TAB 2, 10.1, Samsung Electronics, Seoul, South Korea) [24]; a blood pressure monitor (Model UA-767, plus BT-C, Nonin Medical, Minnesota, USA) [25]; a fingertip pulse oximeter (Nonin, Onyx II 9560, A&D Medical, Tokyo, Japan) [26]; and the Precision Health Scale (UC-321 PBT-C, A&D Medical, Tokyo, Japan) [25]. The Telekit is an asynchronous solution that collects health data from patients at home via an application. The patients use an integrated application, which is available from the tablet. After login to the application, the patients are able to answer disease-related questions, such as:* Do you cough more than usual*? In addition to answering questions, the patients measure their blood pressure, oxygen saturation, heart rate, and weight 1-2 times a week as agreed with their doctor. The blood pressure monitor, fingertip pulse oximeter, and the health scale automatically transfer the measurements via Bluetooth to the application on the tablet. Thereafter, the data are sent to a web portal used by healthcare providers for their interaction with the patients. Using the web portal, healthcare providers receive and analyse the patients' health data on fixed days. The healthcare providers check if the patients' measurements are higher than expected and they also view the patients' answers to disease-related questions. In the light of this information, the healthcare providers can respond quickly and get the patients started on proper treatments [4].  Figure 1 shows the equipment that makes up the Telekit system.

### 2.3. Study Design and Data Collection

In this study, we evaluated the usability of the Telekit system from the Danish TeleCare North trial using an iterative, mixed usability approach. A pretest was conducted during the initial phase of the TeleCare North trial, and a posttest was performed after trial completion. In the pretest, a heuristic evaluation (HE1) and a think-aloud test (TA1) were performed on two consecutive versions (version: 1.5.0, version: 1.11.3) of the Telekit system [20, 21]. In the posttest, the same usability evaluations methods were used to test the latest version (version 1.29.0) of the Telekit system, yielding a second heuristic evaluation (HE2) and a second think-aloud test (TA2). The quantitative results and qualitative findings related to the pre- and posttest were then compared to each other.

#### 2.3.1. Heuristic Evaluation

Heuristic evaluation is an inspection method in which a small set of experts inspect and evaluate a user interface of a system using a list of accepted usability principles (called heuristics). Each expert independently discovers system usability problems by identifying unmet heuristics, that is, heuristic violations, and assesses the severity of each violation. The evaluation produces a list of potential usability problems that may then serve as input for improving the system in a next round of iterations [14, 27].

In this study, the heuristic evaluations (HE1 and HE2) from the pre- and posttest were applied in laboratory settings in which two researchers participated (a moderator and an observer). The moderator's role was to interact with the experts and guide them with respect to operation of the Telekit system if problems occurred or if certain parts of the user interface needed to be explained. The observer's role was to note the experts' comments and to complete a checklist of the heuristic violations in collaboration with the experts.

In each heuristic evaluation, the five experts were asked to perform different representative tasks using the Telekit system. The tasks for both heuristic evaluations included (1)* login*; (2)* read and watch films about the Telekit system*; (3)* read and watch instructions about the Telekit system*; (4)* perform measurements* (blood pressure, heart rate, oxygen saturation, weight, etc.); (5)* find images presenting their measurements*; (6)* write a message to healthcare providers*, and (7)* logout*. Besides these tasks, they were also encouraged to inspect other aspects of the system.

Each expert identified problems in the Telekit system, classified these in accordance with Jakob Nielsen's ten heuristics (Table 1), and scored the severity of each problem (Table 2) [28]. The experts completed a checklist consisting of usability violations and violation severity scores and provided potential solutions. The severity rating scores were based on Rolf Molich's 5-point severity rating scale: 0:* improvements*; 1:* minor problem*; 2:* severe problem*; 3:* critical problem*; and 4:* malfunction* [29]. The total duration of each heuristic evaluation was 90 minutes including introduction, tasks, and debriefing.

#### 2.3.2. Think-Aloud Tests

The goal of performing a think-aloud test is to record potential users' experiences and thoughts about a system. This can be done by giving the users tasks that they have to complete by using the system. The test encourages users to verbalise their thoughts and to express what they are thinking, doing, and feeling when they go through the user interface. The test makes it possible for the observer and moderator to see and understand the cognitive processes that users engage in during task completion [30].

To achieve as real a test setting as possible, the moderator and the observer chose to perform think-aloud tests (TA1, TA2) in the participants' homes. Furthermore, the patient's home provided a safe place and familiar surroundings for the test. The participants were asked to perform the same seven tasks as the experts performed in the heuristic evaluations. It was explained to the participants that the goal of the evaluations was to evaluate the system and not to test their ability to perform tasks. By providing this information, the moderator and the observer hoped that the participants would feel free to comment and criticise the Telekit system. The researchers were present during all the tests. The moderator's role was to interact with the participants, guide them through the tasks, and encourage them to think aloud during the tests. The moderator did not intervene or disrupt the thinking process; only if the participants actively asked for help were they guided to move forward with the system. The observer's role was to record the tests, collect field notes about verbalised and nonverbalised expressions, and furthermore take notes of observations made. In addition, the observer framed a summary of the participants' demographic characteristics (described in Sections 2.1.2 and 2.1.3). The duration of each think-aloud test was approximately 45 minutes including introduction, tasks, and debriefing.

### 2.4. Data Analysis

The data analysis was divided into three parts in order to compare the pre-and posttest of the Telekit system: (1) a pre- and posttest comparison of HE1 versus HE2, (2) a pre- and posttest comparison of TA1 versus TA2, and (3) a posttest comparison of HE2 versus TA2.

The observer's notes from each heuristic evaluation and think-aloud test were computed and transferred to Microsoft Excel 2010. Data from the heuristic evaluation were departed into heuristics, problems, locations, solutions, and severity ratings. To uncover usability issues during the think-aloud tests, the participant comments and moderator observations were counted and categorised into overall usability themes regarding the contents of the identified usability findings. These themes were developed on the basis of discussions and reflections among the observer, the moderator, and the participants. In order to compare data from the mixed usability evaluation methods (HE2 and TA2), usability topics were created from the tasks that the participants and the experts were asked to perform in both usability evaluation methods. The moderator and the observer grouped together the number of identified usability problems during HE2 with the number of usability comments and the number of usability observations encountered in TA2 thereby forming these usability topics.

Spreadsheets were made for both of the heuristic evaluations, regarding distribution of usability problems by heuristic, distribution of the number of usability violations classified into heuristics per expert, and distribution of usability violations into severity degrees among experts. Spreadsheets were also made for both think-aloud tests and used to record the number and contents of usability findings identified by the participants and identified from observer notes. The spreadsheets made it possible to categorise the usability findings into themes and topics and to compare the pretest and the posttest.

## 3. Results

In this section, the results from pre-and posttest of the Telekit system will be presented in three parts: (1) HE1 versus HE2, (2) TA1 versus TA2, and (3) HE2 versus TA2.

### 3.1. Comparison of the Two Heuristic Evaluations (HE1 versus HE2)

In total, the experts identified 152 problems of which 86 (57%) were unique to HE1. In HE2, the experts identified 223 problems of which 101 (45%) were unique. The number of unique usability problems was slightly higher in HE2 than in HE1.

**Figure 2:**
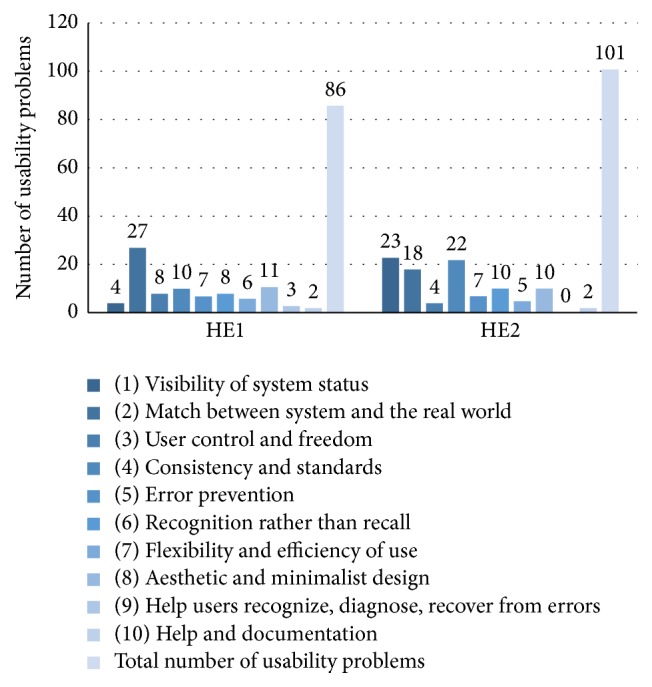
Distribution of usability problems by heuristic for both heuristic evaluations (HE1 and HE2).

Figure 2 presents the distribution of unique usability problems identified for each heuristic in HE1 and HE2, respectively. With the exception of heuristic number (9) (*help users recognize, diagnose, and recover from errors),* all heuristics were used in HE2. The heuristic which had the lowest number of usability problems in HE1 was number (10) (*help and documentation*) with only two (2%) usability problems.

Heuristic number (2) (*match between system and the real world*) with 27 (31%) problems and heuristic number (8) (*aesthetic and minimalist design*) with 11 (13%) problems were associated with the highest number of usability problems in HE1. In comparison with HE2, heuristic number (1) (*visibility of system status*) with 23 (23%) problems and heuristic number (4) (*consistency and standards*) with 22 (22%) problems were associated with the highest number of usability problems.

Table 3 presents the number of usability violations classified into heuristics per expert for the two heuristic evaluations (HE1, HE2). Heuristic number (2) (*match between system and the real world*), heuristic number (4) (*consistency and standards*), and heuristic number (8) (*aesthetic and minimalist design*) were the most referred heuristics during both evaluations. The number of usability violations related to heuristic number (2) (*match between system and the real world)* decreased from 49 in HE1 to 29 in HE2. In contrast, heuristic number (4) (*consistency and standards*) and heuristic number (8) (*aesthetic and minimalist design*) increased from 20 usability violations to 43 and from 20 to 30 in HE2, respectively.

By comparing heuristic number (1) (*visibility of system status*) in HE1 and HE2, an increase from five usability violations in HE1 to 26 usability violations was identified. Heuristic number (7) (*flexibility and efficiency of use*) was associated with eight usability violations in HE1 and 22 usability violations in HE2. Furthermore, in HE2, the number of usability violations relating to heuristic number (10) (*help and documentation*) was three times higher than in HE1.

Table 4 shows the distribution of severity degrees among experts for HE1 and HE2. The severity scores 0, 1, and 2 were the most frequently used in both heuristic evaluations. The severity scores 3 and 4 were used considerably more frequently in HE2 than in HE1, increasing from 4 to 23 the severity score* critical problem* and increasing from 4 to 25 the severity score* malfunction*.

### 3.2. Comparison of the Two Think-Aloud Tests (TA1 versus TA2)

Table 5 is divided into five columns representing the presence of themes (yes/no) in TA1/TA2 and a description of examples of usability findings commented on by the participants during TA1/TA2. The think-aloud tests produced 12 themes: (1)* habits*; (2)* lack of curiosity*; (3)* information level*; (4)* the Telekit system—know how*; (5)* comfortable with the Telekit system*; (6)* usability problems*; (7)* learnability*; (8)* system feedback*; (9)* content of the Telekit system*; (10)* measurements*; (11)* the Telekit design*; and (12)* relevance of the Telekit system*. The columns “Yes” and “No” in the table show which themes were present in TA1 and TA2, respectively. For instance, the theme* information level* was represented in TA2 but not in TA1 and therefore only the TA2 column contains an example/description of the usability finding. Table 5 shows only a selection of the usability problems and therefore the themes can contain several more usability problems than already presented in the table.

Through the participants' verbal and nonverbal language, it became clear that the theme* habits* was relevant for their interaction with the Telekit system. The observer noticed, for example, that during certain tasks the participants avoided using unknown functionalities of the system, and the participants also expressed this stating, “*Why do I have to use that function if the system works fine without it?*” The theme,* lack of curiosity*, was related to the participants' lack of need to build new habits given their existing use of the system. One example of this was a participant who was not interested in using the message functionality because the participant was not used to writing emails. The third theme,* information level*, was relevant because the participants lacked overall knowledge about the Telekit system and their COPD disease. The fourth theme,* the Telekit system—know how*, was related to their experiences with the Telekit system. Some of the participants were surprised to discover certain functionalities, for example, the zoom function or the scroll function.

The fifth theme,* comfortable with the Telekit system*, became relevant because several of the participants expressed that their relatives or doctors had made it clear to them that they could not do anything wrong when interacting with the system and that they could always ask for help if problems occurred. The sixth theme,* usability problems*, was created because several usability problems were identified by the participants during TA1 and TA2. As mentioned in Materials and Methods, before the present study, the participants had received training sessions containing instructions to using the Telekit system. The seventh theme,* Learnability*, was relevant because the majority of the participants reported that they had learned more about how to use the system and had started using some of the functionalities in their daily lives, for example, the functionality allowing them to measure their blood pressure.

The eighth theme,* system feedback*, was based on the technical feedback that participants became acquainted with during the tests. The ninth theme,* contents of the Telekit system*, included formulations, questions, descriptions, and functionalities that were visible in the system. One of the major tasks that participants needed to perform in the Telekit system was to measure their vital signs with different devices. Their ability to do so resulted in the tenth theme,* Measurements*. The eleventh theme,* The Telekit design*, was created by inspiration from Nielsen's heuristic number (8) named* aesthetic and minimalist design*. The theme included design aspects and visual suggestions to improve the system. The last theme,* relevance of the Telekit system*, arose because participants inadvertently verbalised their reflections stating how the Telekit system had suddenly become a very important part of their daily life as it helped them manage their COPD.

### 3.3. Comparison of the Heuristic Evaluation and the Think-Aloud Test (HE2 versus TA2)

In this section, we compare the usability findings identified during the posttest of the Telekit system. We compared the number of usability problems identified in HE2 with the number of usability comments and observations identified by participants and researcher in TA2.  Table 6 below presents the number of problems per expert (E1-E5) classified as follows: (1)* misc.*; (2)* read and watch films*; (3)* read and watch instructions*; (4)* log in*; (5)* perform measurements*; (6)* write a message*; (7)* view images*, and (8)* log out*. The topics were created on the basis of the tasks the experts and participants were asked to perform during the mixed usability evaluation.  Table 6 also illustrates the participants' (P1–P5) number of usability comments by topic and the researcher observations made during TA2. In HE2, the experts identified more usability violations than anywhere else (*n* = 77) in topic 5,* perform measurements*, and the second-largest number of violations was observed for topic 1,* misc.* (*n* = 42). In TA2, the participants and the researcher also had more comments for topic 5,* Perform measurements*, than for any other topic as a total of 25 comments and 12 observations were recorded.

In general, the number of problems identified in HE2 was higher than the number of problems identified in TA2. Overall, there were more expert-identified problems (*n* = 223) than participant comments (*n* = 76) and researcher observations (*n* = 40). Contrary to HE2, in TA2, the participants and the researcher identified the highest number of problems regarding usability topic 8,* log out*. These issues were not reported in HE2 (Table 6).

## 4. Discussion

Recent years have seen a boom in the development of telehealth technologies in the hope that they would save costs for society and provide benefits for patient. Evaluating usability is an important step in the development of telehealth technologies and it is a prerequisite to successful implementation [31–33]. Multiple usability evaluation methods may be used in the testing and evaluation of usability ranging from inspection methods such as heuristic evaluations to user tests such as think-aloud tests.

In the literature, the importance of working iteratively when developing and evaluating systems is often emphasised [19]. Furthermore, experiences and documentation of performed tests describe that using different methods and techniques to assess the usability of a system illuminates different and very important perspectives from the perspectives of both the user and the expert. The various usability methods complement each other, but some methods are preferable to others, depending on how far the system is in the development process. Thus, it is recommendable to combine the methods and to use iterative design processes in which systems are tested repeatedly through circular processes [14].

Some studies have already combined different usability evaluation methods and worked in iterative loops in the design and evaluation of systems [34–36]. Nevertheless, it has not been possible to find studies or other telehealth technologies that use the exact same setup as the present study. However, we did find examples of similar research of other technological systems [37]. For instance, one study has compared two prototypes of a digital emergency medical services system through heuristic evaluations and subsequently examined the validity of the heuristic evaluations in an ethnographic study [38]. Using a mix of usability methods iteratively is important because it allows us to adjust the design of the system. However, when working with usability, it should be remembered that it will always be possible to further improve a system and a “perfect” system will therefore be difficult to attain.

The present study aimed to evaluate the usability of the Telekit system from the Danish TeleCare North trial and to improve its quality and functionalities. Telekit was evaluated by the use of an iterative, mixed usability approach. Specifically, the evaluation included a pre- and posttest of the Telekit system, which uncovered knowledge about the prevalence, severity, and contents of the usability problems. Data were compared descriptively as follows: (1) comparison of HE1 versus HE2; (2) comparison of TA1 versus TA2, and (3) comparison of HE2 versus TA2.

### 4.1. Comparison of HE1 versus HE2

After HE1, Silverbullet received a list of well-documented recommendations to improve Telekit's interface in order to make it more user-friendly. How Silverbullet managed the recommendations was out of the researchers' hands. It is unsure whether the company followed the list to the letter, which is reflected by the usability issues identified through the posttest, HE2 (*n* = 101), and the pretest (*n* = 86). The company's prioritization of usability issues led to the need of further rounds of heuristic evaluations due to the experts' identification of the similar issues supplemented with new issues.

Another explanation could be that the experts had become more aware of any issues in the evaluation process after performing an evaluation of Telekit in the pretest phase. A third explanation may be that three new experts were performing the HE2 assessment instead of the five experts from HE1. From a comparison validity point-of-view, it would have been preferable to use the same experts for the pretest and the posttest. However, the new experts were selected based on the same criteria as the pretest experts in order to achieve the best possible match. These criteria encompassed that the experts should be double experts and have the same education, the same knowledge about the health domain, and the same expertise in the field of usability. However, the use of experts with different levels of familiarity with the assessed system has both pros and cons. The experts who were familiar with the system beforehand may have been affected by their previous experiences with the system. For example, they may have discovered so many errors during the pretest that they had formed a negative, initial opinion which may be difficult to ignore during the second evaluation of the Telekit system. The behaviour of expert number (3) indicates an already negative attitude towards the system, contrary to the negative attitude the expert could have increased the skills in identifying usability issues. Similarly, it may be advantageous to use new experts because they would see and assess the Telekit system with fresh eyes.

### 4.2. Comparison of TA1 versus TA2

The think-aloud tests produced twelve themes and examples of usability findings within these themes. The themes were characterised by both positive and negative findings and results. The majority of the themes appeared in the posttest, which can be explained by the improvements made in the Telekit system in the time between TA1 and TA2. A conflicting explanation may be that changes of the Telekit system after TA1 reduced the usability of Telekit rather than improving it, thus causing more themes to be observed in the posttest.

The think-aloud test was suitable for gaining insight into the COPD participants' thoughts and revealed usability problems encountered during their interaction with the Telekit system. One of the strengths of the think-aloud test is that it allows the collection of data on the users' cognitive processes and how they interact with the system. The test identified both positive and negative aspects of the Telekit system as experienced by the participants. The researchers intended to include the same participants in both tests but finally decided against this because the participants needed to maintain their curiosity and could have generated habits concerning the use of the system during the first test. It was also considered to include time as a result of the think-aloud test. However, it proved too complex to use the time as a measurement element because some parts of the conversations were small talk used to establish an atmosphere in which the participants felt that they were allowed to freely express their opinion. Small talk also helped create a relationship of trust between the participants and the researchers. These talks were deemed necessary since our evaluation of the Telekit system depended on a good relationship.

### 4.3. Comparison of HE2 versus TA2

During the posttest, we chose to perform a comparison across the two different evaluation methods. The Telekit system has undergone many improvements [21], and therefore we compared the results from the second heuristic evaluation (HE2) with the results of the second think-aloud test (TA2). It was desirable to compare the results collected from HE1 and HE2 with the results from TA1 and TA2, but we decided not to do so for the reasons stated above. In addition, data from TA1 and TA2 were not sufficiently comparable to allow for a detailed analysis of these results in comparison with HE1 and HE2 and because the number of participants varied between the pre- and posttest (5 and 6, resp.).

When comparing the results from HE2 and TA2, we found that more usability problems were identified through heuristic evaluation than through think-aloud tests. This may reflect the different scope and potential of the two usability evaluation methods. In contrast to the many problems found using the heuristic evaluation, the positive aspects of the system were not identified by experts but by the COPD participants through the think-aloud test. The COPD participants agreed on some aspects of system usability, but it was clear that they had a different perspective on usability. The experts focused on the system's functionality and interface in general, such as system feedback, navigation, and error prevention. In contrast, the participants focused more on integrating the system into their daily routines, and the themes from the think-aloud test were therefore less technical and more focused on whether the system was meaningful and relevant to them as human beings.

### 4.4. Methodological Considerations

Our results were analysed descriptively, which is an advantage in this type of study because this approach provides an overview of the contents and the scope of the problems. Another reason for performing descriptive analyses was that a large body of material was available for using descriptive analyses, which made it more visually comprehensible. A disadvantage of the descriptive analysis was that statements in relation to the classification of usability problems were not fully exploited by this descriptive type of analysis. However, it was possible to minimise this issue by including two different types of tests representing different perspectives and views on various usability aspects.

The ten heuristics developed by the usability expert Jacob Nielsen and the 5-level severity rating scale given by Rolf Molich were used in both heuristic evaluation sessions [29, 39]. The use of same tool in both sessions enhances the probability of increasing the reliability of the two performed sessions. Many heuristics could have been employed, but by evaluating the Telekit system with the same ten heuristics in the two heuristic evaluation sessions, HE1 and HE2, we gained the possibility of comparing the results of the pretest and the posttest.

The present study has a number of limitations. First, it would have been preferable to use the same experts for both heuristic evaluations which would have made it possible to compare the experts' results individually.

Another limitation is that the pretest included two nonidentical versions of the Telekit system, whereas the versions from the posttest were identical. This occurred because the results from the heuristic evaluation (HE1) led to changes in the Telekit system before the think-aloud test (TA1) had been established. The results from the heuristic evaluation (HE2) were not integrated into the Telekit system before the think-aloud test (TA2) was performed. The advantage of adopting this approach in the pretest phase was that HE1 cleaned the system's interface for potential usability problems which might otherwise have led to lack of interest among users. A disadvantage was that system development took longer time because corrections were made by Silverbullet [23] before new versions of the Telekit system were updated.

Based on the present study, we recommend taking a mixed usability approach and applying it iteratively in the development of medical healthcare systems and telehealth technologies. As an iterative, mixed usability approach was only implemented in a single system, the Telekit system from the Danish TeleCare North trial, we cannot generalise the quantitative results and qualitative findings collected in the present study to other telehealth technologies as the results will likely depend on the type of system, among others.

We adopted a holistic view to evaluate the usability of the Telekit system and followed the system's lifecycle which allowed us to analyse and to elicit the participants' needs and to identify required functionalities. This approach included building a full understanding of both COPD participants' and the experts' perspectives. Developing and evaluating new telehealth systems is an ongoing process. Predicting the duration of this process is no exact science, and when the process ends depends on the users who evaluate the systems and the setting in which the system is going to be implemented.

In the future, it would be interesting to compare the heuristic evaluations and think-aloud tests with a different type of usability evaluation method, such as eye-tracking. Eye-tracking provides objective results in terms of heat maps and subjective findings in the form of comments and statements regarding the heat maps [40]. The objective results and subjective heat maps seem to be a very relevant supplement to the present study because additional usability results may surface that were not identified by the other methods.

## 5. Conclusion

A mixed usability approach performed iteratively on the Telekit system was conducted. Quantitative results and qualitative findings indicated that, to achieve an effective and thorough usability evaluation, it is necessary to combine various methods and to apply these repeatedly. Based on the study results, the Telekit system seems promising as a tool supporting COPD patients in the management of their disease. The heuristic evaluation from the pretest triggered substantial changes in the Telekit system, and several new versions of the system have since been implemented. Thereafter, a think-aloud test was established where participants verbally expressed their experiences with the system. Approximately one year after the system was fully implemented and operational, a posttest was performed. In our comparison of pre- and posttest problems, we assumed that the posttest would identify fewer problems. This assumption was disconfirmed and this illustrates the importance of working iteratively. Furthermore, it is also appropriate to use a mix of usability evaluation methods as each usability method will illuminate different perspectives of usability. The Telekit system still needs functionality and design improvements. However, the system is fully implemented and the COPD patients from the Danish TeleCare North trial have already reported that they experience increased freedom, control, and security and greater awareness of their COPD symptoms when using the system [41]. Hence, we conclude that the Telekit system requires further usability evaluation and recommend mixed methods to be applied in this process. This study was valuable to enhance and customize the Telekit system based on the users' needs by performing extended evaluations and user tests.

## Figures and Tables

**Table 1:** Jakob Nielsen's ten heuristics, including a description of each heuristics.

Jakob Nielsen's ten heuristics	Description of heuristics
(1) Visibility of system status	The system should always keep users informed about what is going on, through appropriate feedback within reasonable time.
(2) Match between system and the real world	The system should speak the users' language, with words, phrases, and concepts familiar to the user, rather than system-oriented terms. Follow real-world conventions, making information appear in a natural and logical order.
(3) User control and freedom	Users often choose system functions by mistake and will need a clearly marked “emergency exit” to leave the unwanted state without having to go through an extended dialogue. Support undo and redo.
(4) Consistency and standards	Users should not have to wonder whether different words, situations, or actions mean the same thing. Follow platform conventions.
(5) Error prevention	Even better than good error messages is a careful design which prevents a problem from occurring in the first place. Either eliminate error-prone conditions or check for them and present users with a confirmation option before they commit to the action.
(6) Recognition rather than recall	Minimize the user's memory load by making objects, actions, and options visible. The user should not have to remember information from one part of the dialogue to another. Instructions for use of the system should be visible or easily retrievable whenever appropriate.
(7) Flexibility and efficiency of use	Accelerators—unseen by the novice user—may often speed up the interaction for the expert user such that the system can cater to both inexperienced and experienced users. Allow users to tailor frequent actions.
(8) Aesthetic and minimalistic design	Dialogues should not contain information which is irrelevant or rarely needed. Every extra unit of information in a dialogue competes with the relevant units of information and diminishes their relative visibility.
(9) Help users recognize, diagnose, and recover from errors	Error messages should be expressed in plain language (no codes), precisely indicate the problem, and constructively suggest a solution.
(10) Help and documentation	Even though it is better if the system can be used without documentation, it may be necessary to provide help and documentation. Any such information should be easy to search, be focused on the user's task, list concrete steps to be carried out, and not be too large.

**Table 2:** Rolf Molich's severity rating scale.

Five-point severity rating scale	Description of severity ratings
(0) Improvement	Which does not substantially disturb the user's experience
(1) Minor problem	The user will be somewhat delayed (few minutes)
(2) Severe problem	The user will be much delayed (several minutes)
(3) Critical problem	The user cannot carry out the task
(4) Malfunction problem	The system does not work properly

**Table 3:** Distribution of the number of usability violations classified into heuristics per expert.

Heuristics	Expert 1		Expert 2		Expert 3		Expert 4		Expert 5		Total	
	HE1	HE2	HE1	HE2	HE1	HE2	HE1	HE2	HE1	HE2	HE1	HE2
(1) Visibility of system status	0	8	0	7	1	4	3	3	1	4	5	26
(2) Match between system and the real world	5	4	8	7	7	4	14	11	15	3	49	29
(3) User control and freedom	1	0	0	3	1	4	3	5	6	2	11	14
(4) Consistency and standards	7	11	3	5	2	18	6	2	2	7	20	43
(5) Error prevention	2	6	3	0	5	8	6	4	2	4	18	22
(6) Recognition rather than recall	2	7	3	9	1	10	2	0	5	1	13	27
(7) Flexibility and efficiency of use	3	5	2	3	0	6	1	4	2	4	8	22
(8) Aesthetic and minimalist design	5	3	3	4	5	19	4	0	3	4	20	30
(9) Help users recognize, diagnose, and recover from errors	2	0	1	0	0	0	0	0	2	0	5	0
(10) Help and documentation	1	1	0	0	0	5	1	3	1	1	3	10

**Table 4:** Distribution of usability violations on Rolf Molich's severity rating scale [29].

Severity degree	Expert 1		Expert 2		Expert 3		Expert 4		Expert 5		Total	
	HE1	HE2	HE1	HE2	HE1	HE2	HE1	HE2	HE1	HE2	HE1	HE2
(1) Improvement	12	16	11	5	10	17	14	2	14	3	61	43
(2) Minor problem	8	19	12	21	12	30	19	11	15	13	66	94
(3) Severe problem	3	6	0	9	0	7	7	8	7	8	17	38
(4) Critical problem	2	3	0	3	0	10	0	5	2	2	4	23
(5) Malfunction	3	1	0	0	0	14	0	6	1	4	4	25

**Table 5:** Usability findings from the think-aloud tests were classified into 12 themes. The table illustrates the themes and the presence of themes (yes/no) and provides examples of the usability findings from TA1 and TA2.

Themes	TA1	TA2	Examples from TA1	Examples from TA2
Habits	Yes	Yes	The users received the Telekit system half a year before and were offered education	The users read the questionnaire very superficially—they know what is going to happen in the subsequent step
Lack of curiosity	Yes	Yes	The users did not attempt to remember password and username	The users are not interested in using the message function for writing or sending messages; the users' curiosity is not aroused
Information level	No	Yes		Users did not use the message menu because they do not know its function
The Telekitsystem—know how	No	Yes		The users know the different icons
Comfortable with the Telekit system	Yes	Yes	The users were satisfied with the functionalities of the Telekit system. The users had no problems with navigation in the Telekit system	The users are not afraid of pressing the wrong key because they know that they always have a way out; the users are comfortable using Telekit
Usability problems	Yes	Yes	The users had difficulties obtaining a reaction from the touchscreen because of cold finger or long nails The users had difficulties remembering username and password which prevented them from logging in	The users did not know how to pause the film It was not easy to find the log-out button
Learnability	No	Yes		The users had no problems with the scroll function
System feedback	Yes	Yes	When the users pressed multiple times on the touchscreen, the Telekit system did not react to commands	The Telekit system's speed was too slow, could be faster
Content of the Telekit system	Yes	Yes	For the experienced users, a more flexible system with les text material would work better	It was nice to be asked about COPD symptoms There was doubt about the meaning of the icons
Measurements	Yes	Yes	When users pressed the touchscreen multiple times, this resulted in incorrect answers and measurements Users made mistakes in the sequences of actions so that the measurements were taken too early or too late because of the location and naming of keys	The users place the blood pressure cuff incorrectly The users start the weight with their feet easily
The Telekit design	Yes	Yes	Some users had difficulties identifying keys on the Telekit keyboard The users had no problems with the scroll function	There was lack of validation when the users change password The typography was appropriate
Relevance of the Telekit system	No	Yes		The users had brought the Telekit system to their doctor

**Table 6:** The eight usability topics, participants' comments per usability, researcher observations, and expert-reported usability violations.

Usability topics within the Telekit system	Researcher observation in TA2						Participant comments in TA2						Expert-reported usability in HE2					
	P1	P2	P3	P4	P5	Total	P1	P2	P3	P4	P5	Total	E1	E2	E3	E4	E5	Total
Misc.	4	1	2	0	1	8	3	6	0	2	2	13	9	7	15	4	7	42
Read and watch films	4	0	0	0	0	4	3	2	0	2	2	9	5	4	11	4	5	29
Read and watch instructions	1	0	0	0	0	1	2	1	2	0	1	6	7	3	7	3	2	22
Log in	1	1	0	1	0	3	2	1	1	0	0	4	4	3	7	1	1	16
Perform measurement	6	3	1	0	2	12	5	5	7	6	2	25	15	16	26	15	5	77
Write a message	4	0	1	0	0	5	1	4	4	3	2	14	4	2	7	3	4	20
View images	3	0	1	1	1	6	2	0	0	0	1	3	1	3	5	2	6	17
Log out	1	0	0	0	0	1	0	0	1	1	0	2	0	0	0	0	0	0

Total	24	5	5	2	4	40	18	19	15	14	10	76	45	38	78	32	30	223
